# Development of infantile tremor syndrome after initiation of hydroxycobalamin treatment in an infant with a late diagnosis of cobalamin C disorder

**DOI:** 10.1002/jmd2.12145

**Published:** 2020-06-26

**Authors:** Ashley Wilson, Vivian Cruz, Jonathan B. Kronick

**Affiliations:** ^1^ Division of Clinical & Metabolic Genetics The Hospital for Sick Children Toronto Canada; ^2^ Department of Pediatrics The University of Toronto Toronto Canada

**Keywords:** cblC, cobalamin C deficiency, combined methylmalonic aciduria and homocystinuria, hydroxycobalamin, vitamin B_12_, movement disorder, infantile tremor syndrome

## Abstract

Combined methylmalonic aciduria and homocystinuria (cobalamin C deficiency, cblC) is a well‐described disorder of vitamin B_12_ metabolism caused by mutations in the *MMACHC* gene with multisystemic manifestations. While there is no cure, combined treatment with intramuscular hydroxycobalamin and oral betaine may reduce the severity of symptoms and improve clinical outcome. We report a female patient diagnosed with late‐onset cobalamin C deficiency at the age of 8 months who presented with developmental regression and severe dermatitis. She developed a movement disorder after initiation of hydroxycobalamin treatment. Similar movement disorders have been described in patients with nutritional vitamin B_12_ deficiencies following cobalamin supplementation but have not previously been reported in patients with cobalamin C disorder. The movement disorder in our patient gradually resolved with clonazepam treatment, despite no seizure activity detected on EEG. She was eventually weaned off the clonazepam and the abnormal movements have not recurred. The patient remains developmentally delayed but is showing no other symptoms related to cobalamin C deficiency. The patient has a younger affected sibling who was treated from birth and who is physically and developmentally entirely normal; she did not have abnormal movements after treatment with hydroxycobalamin was initiated. There is no clear consensus on the cause of movement disorders that develop following initiation of intramuscular vitamin B_12_ treatment.

**SYNOPSIS**

We report on a patient with cobalamin C deficiency who developed a transient movement disorder after initiation of hydroxycobalamin treatment.

## 1 BACKGROUND

Combined methylmalonic aciduria and homocystinuria, cobalamin C type (cblC) (OMIM#277400), is a rare inborn error of vitamin B_12_ metabolism caused by mutations in the *MMACHC* gene (HGNC#24525).^1^ cblC results in impaired intracellular conversion of cobalamin (vitamin B_12_) into adenosylcobalamin and methylcobalamin, biologically active coenzymes required for the metabolism of methylmalonic acid (MMA) and homocysteine.^2^ Biochemically, cblC is typically characterized by elevated MMA, homocysteine, and propionylcarnitine (C3), as well as decreased methionine and total carnitine levels.^3^ Vitamin B_12_ levels are generally normal or mildly elevated. Clinical features of untreated cblC are variable, but typically are multisystemic, including global developmental delay, microcephaly, hypotonia, failure to thrive, seizures, retinopathy, and cardiomyopathy.^4^

Treatment of cblC involves high doses of intramuscular hydroxycobalamin with the aim of enhancing residual enzyme functions in order to lower plasma MMA and homocysteine and normalize plasma methionine.^2^, ^4^, ^5^ Supplementation with oral betaine further reduces homocysteine levels by increasing its conversion to methionine.^2^, ^4^, ^5^ Folate supplementation has not been proven effective, but theoretically may enhance the homocysteine to methionine conversion.^2^ Similarly, supplementation with methionine and/or carnitine have also not been proven as effective treatments, but may be clinically indicated if carnitine and/or methionine levels remain low.^2^, ^3^, ^4^, ^5^ There is no proven effective dietary therapy, and low protein diets should not be used in order to avoid further reduction of methionine levels.^4^, ^5^ With early diagnosis and treatment prior to the onset of symptoms, the effects of the disease can be lessened. cblC is not a targeted disorder for newborn screening in Ontario; however, many patients are diagnosed incidentally via newborn screening due to high C3 levels.

We present a case of a late diagnosed infant with symptomatic cblC who developed infantile tremors after the initiation of high dose hydroxycobalamin treatment. To our knowledge, this apparent complication of hydroxycobalamin treatment has not been previously reported in cblC patients.

## 2 CASE REPORT

An 8 month‐old female was referred to our hospital with a 2 month history of developmental regression, recurrent skin infections, possible seizures, elevated plasma MMA, and elevated homocysteine levels. She was born in Canada at term after a normal pregnancy and delivery. Birth weight was 3823 g. She was the second child born to consanguineous parents of South Asian descent. Her parents and older sister were healthy and well. Newborn screening for metabolic disorders was obtained at 41.5 hours of life and was negative for all screened conditions, which included homocystinuria and methylmalonic acidurias (C3 3.54 μM, cutoff >5.5 μM, C3/C2 ratio 0.20, cutoff >0.23). Her neonatal course was entirely normal and she was discharged home on the fourth day of life. There was no known family history of seizures or metabolic disorders.

Parents reported that the child was developing normally until the age of 6 months, at which time she was admitted to a local hospital for severe diaper dermatitis. Postdischarge, parents noted that the child began developing abnormal movements, to which they attributed to shivering, feeding difficulties, and developmental regression. She was again admitted at the age of 8 months for a recurrence of the diaper dermatitis, at which time a metabolic workup for the developmental regression was initiated. Investigations revealed an elevated plasma C3 level of 3.95 μM (<1.07), elevated plasma homocysteine level at 162.0 μM (5.0‐12.0), and low plasma methionine at 4 μM (11–16). Urine organic acids showed a very large MMA peak at 1100 mmol/mol creatinine. Total and free carnitine levels were 35.1 μM and 24.6 μM, respectively (no reference ranges were available). She did not have hyperammonemia. There was no baseline vitamin B_12_ level; however, there is no dietary reason for it to have been low. The patient was then transferred to our center for workup for a possible cobalamin disorder.

On admission to our hospital the child was nondysmorphic. There was no microcephaly and growth parameters were normal for her age. General physical examination was unremarkable aside from hyperpigmented areas of skin on the arms, legs, neck, and severe diaper dermatitis. Neurological exam revealed significant developmental delay, hypotonia, and hyperreflexia. No tremors, abnormal movements or seizure‐like activity were noted. Mild neutropaenia resolved within 1 day. MRI showed diffuse cerebral atrophy and delayed myelination consistent with changes observed in cblC patients. Ophthalmological, cardiac, and audiology examinations were normal. Initial EEG showed diffuse slow background and intermittent interictal discharges from the right and left temporal head region independently.

Based on these findings and the previous metabolic investigations, she was commenced on immediate treatment with intramuscular injections of hydroxycobalamin at a dose of 1 mg/day, as well as oral l‐carnitine 25 mg/kg/day, betaine 50 mg/kg/day, and folic acid 1 mg/day. Within 8 hours of treatment initiation, the plasma C3 had decreased to 2.97 μM (<1.08) and homocysteine decreased to 38.4 μM (0.5‐11.0). MMA peak on urine organic acids had decreased to 211 mmol/mol creatinine 24 hours after treatment initiation.

On day 6 of admission, the patient developed striking abnormal dyskinetic oromotor and upper limb movements. These movements were involuntary, irregular, and arrhythmic. She progressed to have two episodes of continuous movements with decreased level of consciousness and pinpoint pupils, which only temporarily resolved with multiple doses of lorazepam. A 4‐hour video EEG showed no seizure activity. These dyskinetic movements continued, peaking on day 11 with a significant change in neurological status—the patient lost the ability to fix and follow, and movements became less choreoathetoid and more rhythmic in nature. The patient also experienced three progressively worse clusters of near continuous abnormal movements with right‐sided focality but minimal response to antiepileptic medication loads. These episodes initially resembled status epilepticus. The patient experienced respiratory distress and required CPAP during the third episode and was subsequently transferred to the intensive care unit. Head CT scan showed no evidence of cerebral venous sinus thrombosis (CVST) or acute bleeds. The patient was started on clonazepam (0.05 mg/kg orally) and placed on a 24‐hour video EEG. As with the previous EEG, no seizure activity was captured despite continuous rhythmic twitching of face, eyes and hands, as well as thrashing movements of the arms and head, and grunting. The movements continued over the course of the next week, and gradually resolved with adjustment of clonazepam dosage (increased to 0.1 mg/kg orally).

By day 26, the movements had resolved aside from the occasional mild movement, and the patient was discharged home after 43 days of admission with no movement abnormalities.

Postdischarge she was followed regularly in our metabolic clinic and her parents reported no further abnormal movements. The skin hyperpigmentation and dermatitis resolved with no further recurrence, and the patient started to regain lost developmental milestones. She was successfully weaned off clonazepam at the age of 14 months. DNA and skin biopsy results confirmed a molecular diagnosis of cblC. The patient was found to be homozygous for the pathogenic variant, c.394C>T (p.R132X). As she continued to make developmental progress and had no episodes of metabolic decompensation a subsequent MRI was not obtained.

Currently at the age of 7 years the patient remains significantly developmentally delayed but is making steady gains in her gross motor skills. There has been little progress with fine motor skills and speech. She has begun learning signs. She is not toilet trained. She requires full time support and receives physiotherapy, occupational and speech therapies. She is growing appropriately and eats entirely orally.

## 3 DISCUSSION

The clinical and biochemical features of both untreated cblC and nutritional vitamin B_12_ deficiency are well documented, and the initial metabolic profiles of both conditions can appear similar.^6^ However, while biochemical markers (homocysteine, MMA, C3, and methionine) normalize in patients with nutritional vitamin B_12_ deficiency following initiation of cobalamin supplementation, they improve, but often remain abnormal in patients with cblC. In spite of improvement in the metabolic abnormalities, patients with late treated cblC have variable outcomes and may have persisting developmental delay,^7^, ^8^ as our patient does.

The c.394C>T mutation has typically been documented as resulting in late‐onset cblC^9^, ^10^; however, there have also been several documented cases of patients with homozygous c.394C>T mutations who have presented symptomatically under 1 year of age.^11^, ^12^ Our patient's clinical course of slow, but steady developmental gains aside from intercurrent illnesses is typical of late‐onset disease. While a second disorder cannot be excluded, given our patient's developmental progress, and the other reported cases with the same genotype and infantile presentation, we did not pursue whole exome or whole genome sequencing.

An affected sister was born a few years after the reported patient. Newborn screening for the sister, collected at 39 hours of life did not reveal C3 or C3/C2 levels above the cut off values (C3 2.99 μM, cutoff >5.5 μM; C3/C2 ratio 0.11, cutoff >0.23). Clinical labs collected at birth showed elevated plasma C3 at 1.13 μM (<1.08) and a large MMA peak on urine organic acids of 75 mmol/mol creatinine. Repeat clinical follow‐up at 2 weeks of age again showed an elevated plasma C3 level and a large MMA peak on urine organic acids, as well as a plasma MMA level of 12.47 μM (<0.37), homocysteine level of 39.5 μM (0.5‐11.0), and methionine level of 29 μM (15‐21). She has molecularly confirmed cblC. She has been treated with hydroxycobalamin, betaine, folate and carnitine since diagnosis without any complications, including movement disorders. Her current growth and development at 4 years of age is completely normal.

Several cases of sudden onset movement disorders in infants coinciding with initiation of vitamin B_12_ supplementation in patients with vitamin B_12_ deficiency have been documented in the literature.^6^, ^13^, ^14^, ^15^, ^16^, ^17^, ^18^ The majority of these cases presented with movement disorders within the first 5 days following initiation of intramuscular vitamin B_12_ supplementation. Reported cases are variable in severity, and nature of the movements, and resolution of the movements was usually seen within 2 to 3 weeks. Similar to our patient, most cases did not show any clear seizure activity on EEG, and those whose movement disorders were treated with antiepileptics were typically able to discontinue them upon resolution of the abnormal movements with no recurrence.

The etiology of movement disorders following intramuscular vitamin B_12_ initiation in patients with a vitamin B_12_ deficiency is unclear. Previous reports have suggested imbalances in the B_12_ metabolic pathways due to the rapid change (normalization) in serum B_12_ levels, persistent hyperglycinemia, or exacerbation of a pre‐existing movement disorder as possible causes.^17^, ^18^ However, these hypotheses have not been well studied, and no single hypothesis seems to fit all cases.

Our patient's abnormal movements resembled seizures and thus reporting this apparent complication associated with initiation of parental hydroxycobalamin treatment may be of use in the management of future patients with cblC. It is unknown if patients treated with hydroxycobalamin for other disorders of cobalamin metabolism might also have transient movement disorders upon initiation of treatment.

## CONFLICT OF INTEREST

The authors declare no potential conflict of interests.

## AUTHOR CONTRIBUTIONS

The manuscript was drafted by Ashley Wilson. Jonathan Kronick and Vivian Cruz cared for the patients clinically and revised/reviewed the manuscript. Jonathan Kronick serves as guarantor for the article.

## INFORMED CONSENT

Institutional review board approval was not required for this case report study. Written consent was obtained from the patient's legal guardian for the use of their children's medical information in writing this case report.
